# Prevalence of malocclusions, oral habits and orthodontic treatment need in a 7- to 15-year-old schoolchildren population in Tirana

**DOI:** 10.1186/2196-1042-14-12

**Published:** 2013-06-14

**Authors:** Giuseppina Laganà, Caterina Masucci, Francesco Fabi, Patrizio Bollero, Paola Cozza

**Affiliations:** Department of Orthodontics, University of Rome Tor Vergata (via Montpellier 1), Rome 00133, kragujevac, Italy; Department of Orthodontics, University of Florence (via del Ponte di Mezzo 46–48), Florence, 50127 Italy; Centre for Biostatistics and Bioinformatics (CIBB), University of Rome Tor Vergata, (via Montpellier 1), Rome, 00133 Italy

## Abstract

**Background:**

The aim of present study was to determine the prevalence of malocclusions, oral habits and the need for orthodontic treatment in a sample of 7- to 15-year-old Albanese schoolchildren.

**Methods:**

The final sample comprised 2,617 subjects (1,257 males and 1,360 females), all orthodontically untreated. Occlusal relationship and the functional analysis were recorded for all subjects. The prevalence rates for the dental health component of the index of orthodontic treatment need (IOTN) were calculated. Comparisons between genders were performed for the prevalence of malocclusions, oral habits and IOTN grades (chi-square tests).

**Results:**

Class I, class II and class III malocclusions and asymmetries were observed in 40.4%, 29.2%, 3.2% and 27.1% of the sample, respectively. There were 2,108 subjects (80.6%) that showed oral habits, with females (82.1%) presenting with a greater prevalence rate than males (78.9%). The objective need for orthodontic treatment (grades 4 and 5 of IOTN) was registered in 1,077 subjects (41.2%). This prevalence rate is higher than those reported for other European countries. No significant differences between genders were found for the IOTN grades.

**Conclusions:**

The findings of the present study revealed the need to improve public health plans for orthodontic prevention and screening and to organise the resources in this area in Albania.

## Background

In the last years, orthodontic treatment demand increased in most countries. Epidemiological studies are essential in order to achieve extensive data on the prevalence of malocclusions and of the social need for orthodontic therapy. This information can be used in every country to create public health plans for orthodontic prevention and screening and to organise the resources in this area [1, 2].

Many studies on the prevalence of malocclusions and on the need for orthodontic treatment in different ethnic groups have been published in the last 20 years [1, 3–8]. They reported rather heterogeneous data which reveal a great variability with respect to the design of the survey (i.e. the developmental status of cohort, the clinical examination method, the number of the subjects included in the study) and to the features of the different ethnic groups.

Whereas northern and central European populations have been the object of a great number of surveys [9–14], there are very few investigations that evaluated the prevalence of malocclusions and orthodontic treatment need in southern European ethnic groups [2, 15]. In particular, the Albanese schoolchildren population has never been investigated. However, for every country, it is very important to identify the prevalence of malocclusions and indications for orthodontic treatment in order to improve public oral health [1].

The aim of the present epidemiological study was to assess the prevalence of malocclusions, the prevalence of oral habits and the need for orthodontic treatment by means of the index of orthodontic treatment need (IOTN) [16] in a population of Albanese schoolchildren.

## Methods

### Study population

The study target population consisted of subjects between 7 and 15 years of age who were attending public schools in Tirana (Albania). The fifteen examined schools, ten in the town and five in the province of Tirana, were chosen by the Statistical Department of Teaching Direction of Tirana, using a stratified selection technique, in order to represent the distribution of socio-economic conditions during the school year 2009 to 2010. Classes within schools were sampled systematically, and all students attending the sampled classes were examined. Written consent was obtained from the schoolchildren and their parents before starting examination.

### Final sample

Sample size was calculated assuming a 50% prevalence ratio for any characteristic to be estimated with a 95% confidence interval. This assumption leads to the highest sample size with a precision of 1.9%. A total of 2,707 subjects, 1,302 males and 1,405 females, were randomly selected according to multistage stratified cluster sampling design.

Selection criteria for examination were the presence of deciduous canine and deciduous second molar in primary dentition and mixed dentition, the presence of permanent canine and first molar in permanent dentition and no history of orthodontic treatment. Exclusion criteria for this study were subjects with craniofacial anomalies (syndromes), subjects with no Albanese citizenship and students with past or present history of orthodontic treatment.

After the application of both inclusionary and exclusionary criteria, the final sample consisted of 2,617 subjects, 1,257 males and 1,360 females.

### Clinical examination

The orthodontic examination was carried out by five examiners. Before clinical registration, they took part in a course on methods of clinical research and orthodontic diagnosis. A pilot study on 50 children was conducted before beginning the present investigation to ensure the accuracy of diagnosis and to standardise the procedures; no statistically significant differences were found (*P* > 0.05).

The schoolchildren were examined in the medical room of the schools. The examination lasted 20 min per child, following the WHO guidelines [17]. Occlusal conditions were assessed using latex gloves, mouth mirrors, callipers and millimetre rulers.

For each subject, a registration chart was designed. It comprised an anamnestic questionnaire and clinical examination measurements without radiograms.

### Orthodontic variables

The following parameters were evaluated during the examination:

- *Occlusal relationships: canine and molar sagittal relationships (according to Angle's classification)*[18]*and coincidence of incisor midlines*. Findings were classified in the following categories: class I, class II divisions 1 and 2 and class III malocclusions. Occlusal patterns of patients that deviated from the class I relationship (including crowding, spacing and rotations) were categorised as class I malocclusion.

In the group ‘class I malocclusion’, subjects presenting with bilateral canine and molar class I relationship (permanent dentition) or neutroclusion (mixed dentition) with crowding or other dental malpositions were included. The group ‘class II malocclusion’ consisted of subjects presenting with bilateral canine and molar class II relationship (divisions 1 and 2). The group ‘class III malocclusion’ consisted of subjects presenting with bilateral canine and molar class III relationship. In the group ‘Asymmetries’, subjects with a different relationship on both sides of the occlusion and subjects presenting facial asymmetries were included.

- *Functional analysis*[19]*: swallowing, breathing, sucking habits, nasal obstruction and speech defects*.

The need for orthodontic treatment was assessed for each subject by means of the dental health component (DHC) of the IOTN [16]. This index presents five grades of different need of treatment: grades 5 and 4 represent high priority of treatment, grade 3 represents borderline treatment and grades 2 and 1 represent little or no need for treatment (Table 1[16]).

**Table 1:** **Grades of the dental health component of the index of orthodontic treatment need**

Grade		Description
Grade 5	Very great	Defects of cleft lip and/or palate; increased overjet greater than 9 mm; reverse overjet greater than 3.5 mm with reported masticatory or speech difficulties; impeded eruption of teeth (with the exception of third molars) due to crowding, displacement, the presence of supernumerary teeth, retained primary teeth and any other pathological cause; extensive hypodontia with restorative implication (more than one tooth missing in any quadrant) requiring pre-restorative orthodontics
Grade 4	Great	Increased overjet greater than 6 mm but less than or equal to 9 mm; reverse overjet greater than 3.5 mm with no reported masticatory or speech difficulties; reverse overjet greater than 1 mm but less than or equal to 3.5 mm with reported masticatory or speech difficulties; anterior or posterior crossbites with greater than 2 mm displacement between retruded contact position and intercuspal position; posterior lingual crossbites with no occlusal contact in one or both buccal segments; severe displacement or teeth greater than 4 mm; extreme lateral or anterior open bite greater than 4 mm; increased and complete overbite causing notable indentation on the palate or labial gingivae; patient referred by colleague for collaborative care, e.g. periodontal, restorative or TMJ considerations; less extensive hypodontia requiring pre-restorative orthodontics or orthodontic space closure to obviate the need for a prosthesis (not more than one tooth missing in any quadrant)
Grade 3	Moderate	Increased overjet greater than 3.5 mm but less than or equal to 6 mm with incompetent lips at rest; reverse overjet greater than 1 mm but less than or equal to 3.5 mm; increased and complete overbite with gingival contact but without indentations or signs of trauma; anterior or posterior crossbites with less than or equal to 2 mm but greater than 1 mm displacement between retruded contact position and intercuspal position; moderate lateral or anterior open bite greater than 2 mm but less than or equal to 4 mm; moderate displacement of teeth greater than 2 mm but less than or equal to 4 mm
Grade 2	Little	Increased overjet greater than 3.5 mm but less than or equal to 6 mm with competent lips at rest; reverse overjet greater than 0 mm but less than or equal to 1 mm; increased overbite greater than 3.5 mm with no gingival contact; anterior or posterior crossbites with less than or equal to 1 mm displacement between retruded contact position and intercuspal position; small lateral or anterior open bites greater than 1 mm but less than or equal to 2 mm; pre-normal or post-normal occlusions with no other anomalies; mild displacement of teeth greater than 1 mm but less than or equal to 2 mm
Grade 1	None	Other variation in occlusion including displacement less than or equal to 1 mm

### Statistical methods

Data were registered in Excel 2007 (Microsoft, Redmond, WA, USA) and elaborated using the Statistical Package for the Social Sciences Windows, version 15.0 (SPSS, Chicago, IL, USA). Descriptive statistics were calculated for every measured variable and for DHC grades of the IOTN in order to evaluate the studied sample. Categorical variables were analysed using the chi-square test of Pearson to determine differences in prevalence rates between genders. *P* value for statistical significance was set at 0.05.

## Results and discussion

### Results

A total of 2,617 subjects, 1,257 males (48.4%) and 1,360 females (51.6%) 7- to 15-year-old schoolchildren, were examined. The studied sample represented 3% of the total population.

In Table 2 the composition of the sample by age and gender is described. Table 3 shows the distribution of the sample according to the prevalence of malocclusions. Findings indicated that the subjects presenting with class I malocclusion were 1,058 (40.4% of the total sample), the subjects showing class II malocclusion were 764 (29.2%) and the subjects having class III malocclusion were 85 (3.2%); asymmetries were present in 710 subjects (27.1%). No statistically significant differences with regard to distribution of malocclusions were found between genders (chi-square = 4.350, *P* = 0.226; Table 4).

**Table 2:** **Composition sample (** ***n*** **= 2,617) by age and gender**

Age (year)	Total sample			Composition sample by age	Composition sample by gender	
	Males	Females	Males + Females	Males + Females (%)	Males (%)	Females (%)
	***n***	***n***	***n***			
7	132	137	269	10.3	49.1	50.9
8	101	140	241	9.2	41.9	58.1
9	143	145	288	11.0	49.7	50.3
10	127	145	272	10.4	46.7	53.3
11	136	151	287	11.0	47.4	52.6
12	140	153	293	11.2	47.8	52.2
13	164	142	306	11.7	53.6	46.4
14	167	179	346	13.2	48.3	51.7
15	147	168	315	12.0	46.7	53.3
Total	1,257	1,360	2,617	100.0	48.4	51.6

**Table 3:** **Prevalence of malocclusions in the total sample (** ***n*** **= 2,617)**

Malocclusions	Number	Percentage	Confidence interval inferior (%)	Confidence interval superior (%)
Class 1	1,058	40.4	37.4	43.4
Class 2	764	29.2	26.0	32.4
Class 3	85	3.2	−0.5	6.9
Asymmetries	710	27.1	23.8	30.4
Total	2,617	100.0	100.0	100.0

**Table 4:** **Prevalence of malocclusions by gender**

Malocclusions by gender	Class 1	Class 2	Class 3	Asymmetries	Total
	***n***(%)	***n***(%)	***n***(%)	***n***(%)	***n***(%)
Males	508 (40.4)	347 (27.6)	45 (3.6)	357 (28.4)	1,257 (100.0)
Females	550 (40.4)	417 (30.7)	40 (2.9)	353 (26.0)	1,360 (100.0)

The prevalence of malocclusions by age is described in Table 5. Oral habits (Table 6) were present in 2,108 subjects (80.6%), 992 males (78.9%) and 1,116 females (82.1%), with subjects showing more than one habit. Among the oral habits, the highest prevalence rate was registered for pacifier habit (30%), which was followed by oral breathing (23.2%) and atypical swallowing (16.2%). Statistically significant differences were found between males and females for oral habits (*P* = 0.048; Table 7), with female subjects showing a greater prevalence rate than male subjects (82.1% vs 78.9%). In particular, finger sucking habit (*P* = 0.001) showed a significantly greater prevalence rate in male subjects (12.3% vs 8.3%), while pacifier habit (*P* = 0.044) showed a significantly greater prevalence rate in female subjects (31.8% vs 28.1%).

**Table 5:** **Prevalence of malocclusions by age (7 to 15 years)**

Malocclusions by age (years)	Class 1	Class 2	Class 3	Asymmetries	Total
	***n***(%)	***n***(%)	***n***(%)	***n***(%)	***n***(%)
7	161 (15.2)	50 (6.5)	6 (7.0)	52 (7.3)	269 (10.3)
8	112 (10.6)	61 (8.0)	10 (11.8)	58 (8.2)	241 (9.2)
9	132 (12.5)	71 (9.3)	10 (11.8)	75 (10.6)	288 (11.0)
10	112 (10.6)	76 (9.9)	11 (12.9)	72 (10.1)	271 (10.3)
11	95 (9.0)	105 (13.7)	11 (12.9)	76 (10.7)	287 (11.0)
12	112 (10.6)	86 (11.2)	5 (5.9)	90 (12.7)	293 (11.2)
13	108 (10.2)	103 (13.4)	8 (9.4)	87 (12.2)	306 (11.7)
14	125 (11.8)	112 (14.6)	10 (11.8)	99 (13.9)	346 (13.2)
15	101 (9.5)	100 (13.0)	13 (15.3)	101 (14.2)	315 (12.0)
Total	1,058 (100.0)	764 (100.0)	85 (100.0)	710 (100.0)	2,617 (100.0)

**Table 6:** **Prevalence and distribution of oral habits in the total sample**

Oral habits	Number	Percentage	Confidence interval inferior (%)	Confidence interval superior (%)
Any habits	2,108	80.6	79.1	82.1
Finger	268	10.2	9.0	11.4
Pacifier	785	30.0	28.2	31.8
Mouth breathing	607	23.2	21.6	24.8
Atypical swallowing	424	16.2	14.8	17.6
Low tongue position	250	9.6	8.5	10.7
Lip interposition	104	4.0	3.2	4.7
Other habits	441	16.9	15.5	18.3

**Table 7:** **Prevalence and distribution of oral habits by gender**

Oral habits	Males	Females	Chi-square test	
	***n***(%)	***n***(%)	Chi-square	***P***
Any habits	992 (78.9)	1,116 (82.1)	3.915	*0.048*
Finger	155 (12.3)	113 (8.3)	11.063	*0.001*
Pacifier	353 (28.1)	432 (31.8)	4.044	*0.044*
Mouth breathing	309 (24.6)	298 (21.9)	2.467	0.116
Atypical swallowing	80 (6.3)	99 (7.3)	0.721	0.396
Low tongue position	221 (17.6)	265 (19.5)	1.442	0.230
Lip interposition	42 (3.3)	63 (4.6)	2.502	0.114
Other habits	204 (16.2)	237 (17.4)	0.586	0.444

Table 8 shows the prevalence rates of the IOTN grades in the whole sample. An objective treatment need (grade 5 and grade 4) was recorded in 1,077 subjects: grade 5 was registered in 101 subjects (3.9% of the schoolchildren), and grade 4 was registered in 976 subjects (37.3%). Borderline need, grade 3, was observed in 847 subjects (32.4%). Little need for orthodontic treatment (grade 2) was reported for 386 subjects, which is 14.7% of the schoolchildren. Only 307 subjects, 11.7% of the studied population, presented no need for orthodontic treatment (grade 1). No statistically significant differences were reported between genders for any grade of DHC of the IOTN (Table 9). Table 10 shows the prevalence rates of the grades of DHC of the IOTN by age.

**Table 8:** **Dental health component of the IOTN: prevalence in the total sample (** ***n*** **= 2,617)**

IOTN grade	Number	Percentage	Confidence interval inferior (%)	Confidence interval superior (%)
1	307	11.7	8.1	15.3
2	386	14.7	11.2	18.3
3	847	32.4	29.2	35.5
4	976	37.3	34.3	40.3
5	101	3.9	0.1	7.6
Total	2,617	100.0	100.0	100.0

**Table 9:** **Dental health component of the IOTN: frequencies by gender**

IOTN	Males	Females	Chi-square test	
	***n***(%)	***n***(%)	Chi-square	***P***
1	133 (10.6)	174 (12.8)	2.880	0.090
2	188 (15.0)	198 (14.6)	0.053	0.817
3	417 (33.2)	430 (31.6)	0.654	0.419
4	464 (36.9)	512 (37.6)	0.121	0.728
5	55 (4.4)	46 (3.4)	1.479	0.224

**Table 10:** **Dental health component of the IOTN: frequencies by age**

IOTN	1	2	3	4	5	Total
	***n***(%)	***n***(%)	***n***(%)	***n***(%)	***n***(%)	***n***(%)
7	45 (14.7)	53 (13.7)	97 (11.5)	66 (6.8)	8 (7.9)	269 (10.3)
8	20 (6.5)	44 (11.4)	97 (11.5)	73 (7.5)	7 (6.9)	241 (9.2)
9	29 (9.4)	59 (15.3)	83 (9.8)	110 (11.3)	7 (6.9)	288 (11.0)
10	27 (8.8)	43 (11.1)	90 (10.6)	100 (10.2)	12 (11.9)	272 (10.4)
11	28 (9.1)	25 (6.5)	92 (10.9)	135 (13.8)	7 (6.9)	287 (11.0)
12	35 (11.4)	47 (12.2)	95 (11.2)	106 (10.9)	10 (9.9)	293 (11.2)
13	44 (14.3)	31 (8.0)	92 (10.9)	124 (12.7)	15 (14.9)	306 (11.7)
14	48 (15.6)	40 (10.4)	98 (11.6)	143 (14.7)	17 (16.8)	346 (13.2)
15	31 (10.1)	44 (11.4)	103 (12.2)	119 (12.2)	18 (17.8)	315 (12.0)
Total	307 (100.0)	386 (100.0)	847 (100.0)	976 (100.0)	101 (100.0)	2,617 (100.0)

### Discussion

The present study represents the first epidemiological survey carried out on an Albanian population with the primary aim to achieve a true image of the orthodontic conditions of the Albanian students aged 7 to 15 years. The study sample was evaluated in order to assess the prevalence of malocclusions, to report the prevalence of oral habit and to record the orthodontic treatment need by means of the DHC of the IOTN. With regard to the occlusal findings, class I malocclusion was found in 40.4% of the examined population, class II malocclusion was found in 29.2% and class III malocclusion was found in 3.2% of the subjects. Dental asymmetries were recorded in 27.1% of the study sample. Therefore, more than one third of the sample presented with a class I malocclusion. The distribution of the different types of dental malocclusions is mildly different from the outcomes of other European epidemiologic surveys [2, 11, 13]. Lux et al. [14] in 2009 reported similar prevalence rates of sagittal dental relationship for a sample of German schoolchildren aged 9 years. Quite comparable data could be found in two studies carried out in France and in Iceland with the aim of describing the orthodontic conditions in samples of subjects presenting with deciduous dentition [9, 10]. The comparison between the Albanian sample evaluated in the present survey and different ethnic groups showed different prevalence rates of malocclusions with respect to Latino-American populations [5, 6], while for a sample of Iranian adolescents, quite similar outcomes were reported [1]. These findings can be used as reference data for the proper description of the epidemiology of malocclusions on a wide spectrum of different ages within which it is possible to identify the optimal timing for treatment of many dentoskeletal disharmonies [2].

By means of the anamnestic questionnaire and the functional analysis, the prevalence of oral habits was assessed in the study sample. A total of 2,108 subjects (80.6% of the total sample) showed oral habits, with subjects presenting with more than one habit. The findings suggest that the most frequent oral habit was pacifier habit, which was observed in 785 subjects (30.0% of the total sample), followed by oral breathing that was recorded in 23.2% of the schoolchildren and by atypical swallowing that was found in 16.2% of the subjects. The prevalence of oral habits in male and in female samples was similar (78.9% and 82.1%, respectively), though it was recorded as a statistically significant difference. Finger sucking habit was significantly more prevalent in male subjects, while the pacifier habit was more frequent in female subjects.

The total prevalence rate for oral habits is quite comparable to the data reported in other surveys, such as the study by Aznar et al. in 2006 [20] on a sample of children aging 3 to 6 years and the study by Hebling et al. in 2008 [21] on a sample of 5-year-old children. The Albanian population showed lower prevalence rates for the pacifier habit and the finger sucking habit than an Italian sample of 6-year-old children [22].

In the present study, the DHC of the IOTN, which is considered an objective and synthetic method [2], was used to assess the need for orthodontic treatment. According to the index, 11.7% of the total sample needs an orthodontic treatment for very severe malocclusions (grade 5). The prevalence rate (41.2%) of Albanese children assigned to grades 4 and 5 (objective need for orthodontic treatment) is higher than the prevalence rate recorded in European subjects of other populations assigned to the same grades using the DHC of the IOTN. In a British sample of 12- to 15-year-old subjects, 21% to 35% was judged to have a definite orthodontic treatment need [12]. Souames et al. in 2006 [23] found for the French population an objective need for orthodontic treatment in 21% of the sample (grades 4 and 5). Two southern European samples of schoolchildren (a Spanish sample and an Italian sample) [2, 15] showed very low percentages of subjects assigned to grade 4 or 5 of the DHC (21.8% to 17.1% and 27.3%, respectively) when compared with the Albanian sample. On the contrary, the findings of the present survey can be assimilated to the outcomes of some studies carried out on populations from Asian or African countries. Indeed, Abdullah and Rock, in 2001 [4], in their study on a Malaysian sample of 12- to 13-year-old schoolchildren reported a prevalence for an objective orthodontic treatment need of 47.9%. Likewise, 42.6% of a Senegalese sample of 12- to 13-year-old adolescents was assigned to grade 4 or 5 of the DHC of the IOTN [8]. These findings could be explained considering the very hard social and economic conditions in which these populations live. On the other hand, the lower prevalence rate for definite orthodontic need reported in the mentioned epidemiological European surveys on adolescents could be caused by appropriate preventive measures or interceptive orthodontic treatment performed at an early age. As for the outcomes of the present study, the data refer to a very large sample of subjects between 7 and 15 years of age, which represent a very heterogeneous sample; this sample include subjects at an early stage of growth who, in other European countries, probably would undergo an interceptive orthodontic treatment [24–26]. These considerations might suggest that preventive strategies and early orthodontic treatment, adopted in other countries, could be successfully integrated in the development of an effective national programme in Albania aimed at increasing the level of oral health and reducing malocclusion risk factors.

## Conclusions

The following conclusions were drawn from this study:

- Class I malocclusion was observed in 40.4% of the total sample, class II was observed in 29.2%, class III was observed in 3.2% and asymmetries were observed in 27.1%.
- Oral habits were registered in 80.6% of the sample with female subjects showing a greater prevalence rate. The most frequent oral habit was pacifier habit observed in 785 subjects (30.0% of the total sample).
- The prevalence rates for grade 5 and grade 4 of the DHC of the IOTN in the total sample were 3.9% and 37.3%, respectively.
- The findings of the present study indicate the real need to improve and integrate preventive strategies in a national programme in Albania in order to reduce malocclusion risk factors.
